# Use of antidepressants among Finnish family caregivers: a nationwide register-based study

**DOI:** 10.1007/s00127-021-02049-1

**Published:** 2021-03-01

**Authors:** Tuija M. Mikkola, Hannu Kautiainen, Minna Mänty, Mikaela B. von Bonsdorff, Hannu Koponen, Teppo Kröger, Johan G. Eriksson

**Affiliations:** 1 Folkhälsan Research Center, PO Box 211, 00251 Helsinki, Finland; 2 Clinicum, Faculty of Medicine, University of Helsinki, Helsinki, Finland; 3 Primary Health Care Unit, Kuopio University Hospital, Kuopio, Finland; 4 Unit of Strategy and Research, City of Vantaa, Vantaa, Finland; 5 Department of Public Health, University of Helsinki, Helsinki, Finland; 6 Gerontology Research Center and Faculty of Sport and Health Sciences, University of Jyväskylä, Jyväskylä, Finland; 7 Department of Psychiatry, University of Helsinki and Helsinki University Hospital, Helsinki, Finland; 8 Department of Social Sciences and Philosophy, University of Jyväskylä, Jyväskylä, Finland; 9 Singapore Institute for Clinical Sciences, Agency for Science, Technology, and Research, Singapore, Singapore; 10 Department of Obstetrics and Gynaecology and Human Potential Translational Research Programme, Yong Loo Lin School of Medicine, National University of Singapore, Singapore, Singapore; 11 Department of General Practice and Primary Health Care, University of Helsinki, Helsinki, Finland

**Keywords:** Informal caregiving, Ageing, Antidepressants, Mental health, Registers

## Abstract

**Purpose:**

The purpose of this study was to compare the use of antidepressants over 6 years between family caregivers providing high-intensity care and a matched control population using register-based data.

**Methods:**

The study includes all individuals, who received family caregiver’s allowance in Finland in 2012 (*n* = 29,846 females, mean age 66 years; *n* = 12,410 males, mean age 71 years) and a control population matched for age, sex, and municipality of residence (*n* = 59,141 females; *n* = 24,477 males). Information on purchases of antidepressants, including the number of defined daily doses (DDD) purchased, between 2012 and 2017 was obtained from the national drugs reimbursement register.

**Results:**

During the follow-up, 28.5% of female caregivers and 23.5% of the female controls used antidepressants, while the numbers for males were 21.1% and 16.4%, respectively. Adjusted for socioeconomic status, female caregivers used 43.7 (95% confidence interval 42.4–45.0) and their controls used 36.2 (35.3–37.2) DDDs of antidepressants per person-year. Male caregivers used 29.6 (27.6–31.6) and their controls used 21.6 (20.2–23.0) DDDs of antidepressants per person-year. Among female caregivers, the relative risk for use of antidepressants was similar (about 1.3) from 20 to 70 years, after which the relative risk declined. In male caregivers, the relative risk was highest (about 1.4–1.5) between 45 and 65 years.

**Conclusions:**

Family caregivers providing high-intensity care use more antidepressants and hence, are likely to have poorer mental health than the age-matched general population in virtually all age groups. However, the magnitude of the higher use varies as a function of age and gender.

## Introduction

Family caregiver is a person, who takes care of a relative or loved one because of an illness, disability or other specific need for care. The need of family caregiving is increasing because of an aging population and because improved healthcare results in an increase in life expectancy also among those with chronic diseases and disabilities. Without family caregivers, the economic burden of long-term care for societies would be overwhelming [1, 2]. However, family caregiving may be demanding, and family caregivers may pursue care tasks with a cost to their own well-being. Time-consuming and demanding care responsibilities may lead to chronic stress and social isolation, which increase the risk of depression [3]. Mental health problems of a caregiver may, in turn, threaten continuity and quality of care [4].

A number of studies have investigated mental health of caregivers. However, most of the studies lack a non-caregiving control population [5, 6], use small convenience samples (for review see [7]), focus only on caregivers of a specific care recipient group [7–9], or on caregivers of specific age [9–14], usually older caregivers. There are only a few larger population-based surveys, which have studied the association between family caregiving and self-reported mental health and they have generally reported poorer mental health in caregivers than in non-caregivers [15–17]. Two studies based on Northern Ireland Census 2011 reported a lower level of self-reported mental health problems among low-intensity family caregivers compared to non-caregivers but a higher level of these problems in high-intensity family caregivers [18, 19]. Family caregivers have a large age spectrum, but the moderating effects of age and gender on caregiver’s mental health have been little studied. To identify risk groups among caregivers, it is important to know whether age and gender influence the risk of mental health problems among caregivers.

National administrative registries are an ample source of objective health related data. Surveys often have less than optimal response rate raising a question about selection bias, while one advantage of national registries is that they are comprehensive, typically including all residents. To the best of our knowledge, only one previous large study has utilized register-based data to study the associations between caregiving status and use of psychoactive drugs [19]. That study examined antidepressant prescriptions in Northern Ireland but the study was limited by the short follow-up time, 2 years, and by the fact that the information on antidepressant prescriptions was partly collected from the year preceding determination of caregiving status.

The purpose of the present study was to compare use of antidepressants during 6 years between family caregivers, who provide high-intensity care, and a matched control population using register-based data separately for men and women. The moderating effect of age on the differences in use of antidepressants between caregivers and the control population was also analysed.

## Methods

### Material

The study included all individuals in Finland, who were officially recognized family caregivers (‘caregiver’ herein after) in 2012 based on a record of receiving family caregiver’s allowance. According to Act on Support for Informal Care, family caregiver’s allowance can be granted by Finnish municipalities to a person, who provides care or attendance at home due to care recipient’s functional limitation, illness, disability or other comparable reason. A such caregiver is typically the spouse or a parent of the care receiver [20]. Granting of family caregiver’s allowance depends on the intensity of care needed by the care recipient but does not depend on the family caregiver’s income or employment status. These family caregivers can be considered to provide high-intensity care as 69% of the respondents of a Finnish survey for the recipients of family caregiver’s allowance in 2012 reported providing care for 13–24 h per day and 16% reported providing care for 7–12 h per day [21]*.*

All individuals, who had registered income in the Tax Administration’s category “Family caregiver’s or private caregiver’s allowance” in 2012 were identified. Next, private caregivers could be excluded based on information on receipt of private caregiver’s tax deductions, because family caregivers are not entitled to these tax deductions. Altogether, 42,372 family caregivers were identified. Of these caregivers, further register information could not be retrieved for 104 individuals (two with erroneous personal identity code, 102 had forbidden the disclosure of their personal information for safety reasons). Eight caregivers had died before January 1, 2012 and were thus excluded. Four caregivers were removed, because they were considered as being in institutional care. The final number of caregivers in the analyses was 42,256 (about 1% of the adult population in Finland).

Two controls matched according to year of birth, sex, and municipality of residence (index date January 1, 2012) per one caregiver were drawn without replacement from the register of the Population Register Centre. For 28 caregivers, only one matching control subject was found and for 16 caregivers no matching control subjects were found. After removing individuals, who were in institutional care according to the information obtained from the national Care Register for Social Welfare (administered by the National Institute for Health and Welfare), the final number of controls was 83,618.

Data linkages were performed by register-keeping authorities using personal identity codes. These authorities pseudonymised the data. The study plan was approved by the Ethics Committee of the Helsinki and Uusimaa Health Care District (HUS/1955/2018).

### Use of antidepressants

Information on all reimbursed antidepressant (Anatomical Therapeutic Chemical [ATC] code N06A) purchases, including date of purchase, number of defined daily doses (DDD) purchased, and ATC code, were obtained from the register of the Finnish Social Insurance Institution (SII) for the years 2012–2017. DDD is the assumed average maintenance dose per day for a drug used for its main indication in adults and is a useful unit of measurement in pharmacoepidemiology [22]. In absence of information on the actual doses prescribed to the subjects, we used DDD as the estimated daily dose and the number of DDDs as an estimate for the duration of the pharmacotherapy. Number of DDDs can be derived using information on the strength of the product, the number of units in the package(s) purchased, and the DDD of the substance. Using DDDs, information on different types of medicinal substances with different dosages can be combined. The SII register contains pharmacy claims on all prescription drug purchases reimbursed to Finnish residents in non-institutional settings. First, we created a dichotomous variable, having used antidepressants during the follow-up (at least one purchase of antidepressants, yes/no). Second, the total number of DDDs used during the 6-year follow-up was calculated for each individual.

### Follow-up time

Follow-up time was calculated in person-years as the difference between January 1, 2012 and either the date of moving abroad or the date of death or December 31, 2017, whichever occurred first. The dates of moving abroad were obtained from the Population Register Centre, and the dates of death from the Finnish Causes of Death Register maintained by Statistics Finland.

### Other variables

Information on birth year was obtained from the Population Register Centre. Age at baseline was calculated as 2012 minus the birth year. Years of education were calculated based on the highest degree attained by 2012, obtained from Statistics Finland. Information on the annual wage income, caregiver’s allowance, and capital income was retrieved from the register of the Finnish Tax Administration. For descriptive purposes, employment status in 2012 was derived based on the information on socioeconomic position obtained from Statistics Finland [23] and income information. Socioeconomic position was re-categorised into employment status including three categories (1) employed/student (2) unemployed/employed part-time (3) pensioner. A person was classified as unemployed/employed part-time if s/he was unemployed or if the socioeconomic position was unknown and annual earned income was less than 9000 € per year. Those with both unknown socioeconomic position and annual earned income 9000 € or more per year were classified as employed.

### Statistical analysis

All analyses were stratified according to gender. For the analyses, a new, continuous socioeconomic status (SES) variable was computed based on years of education and total income to overcome the spurious effect resulting from the mutual associations between age, years of education and income; older adults have fewer years of education than younger adults and there is a drop in income with age at the time of retirement. Van der Waerden rank-based normalization [24] was used to yield standardized scores for each of the two variables (education years and income) and then, the average of these scores was computed. Logistic regression models were used to derive proportions of antidepressant users among caregivers and controls, adjusted for SES. Poisson regression models adjusted for SES were used to compare the numbers of DDDs of antidepressants used per person-year between caregivers and controls. Finally, restricted cubic spline logistic regression models with 4 knots and adjusted for SES were used to derive caregiver’s relative risk of for antidepressant use as a function of age at baseline. The knots were located at the 5th, 35th, 65th, and 95th percentiles of age based on Harrell’s recommended percentiles [25]. Stata 16.0 (StataCorp LP; College Station, Texas, USA) statistical package was used for the analyses.

## Results

Male caregivers were older than female caregivers (Table 1) . Both male and female caregivers were less educated than their controls. Among both male and female caregivers, the proportion of those working or studying was lower than the proportion among their controls. The proportion of pensioners was higher among male caregivers than among female caregivers. Income was higher in male than in female caregivers. Both female and male caregivers had higher total income (caregiver’s allowance included) than their controls.

**Table 1:** Background characteristics of the female and male family caregivers and their controls at baseline in 2012

	Female (*n* = 88,987)		Male (*n* = 36,887)	
	Control (*n* = 59,141)	Caregiver (*n* = 29,846)	Control (*n* = 24,477)	Caregiver (*n* = 12,410)
Age, years mean (SD)	65.4 (16.0)	65.6 (16.1)	70.1 (15.1)	70.5 (15.4)
Education, mean years (SD)	12.2 (2.8)	11.8 (2.6)	11.8 (2.8)	11.5 (2.6)
Employment status, *n* (%)				
Employed/student	21,128 (35.7)	9447 (31.7)	5860 (23.9)	2296 (18.5)
Unemployed/employed part-time	2191 (3.70)	1709 (5.7)	986 (4.0)	710 (5.7)
Pensioner	35,822 (60.6)	18,690 (62.6)	17,631 (72.0)	9404 (75.8)
Income in euros, median (IQR)				
Total	19,551 (12,820–30,389)	19,669 (14,531–28,781)	22,125 (14,144–35,109)	22,808 (16,484–32,355)
Without caregiver’s allowance	19,551 (12,820–30,389)	15,079 (10,315–24,384)	22,125 (14,144–35,109)	18,511 (12,452–27,925)

**Fig. 1:**
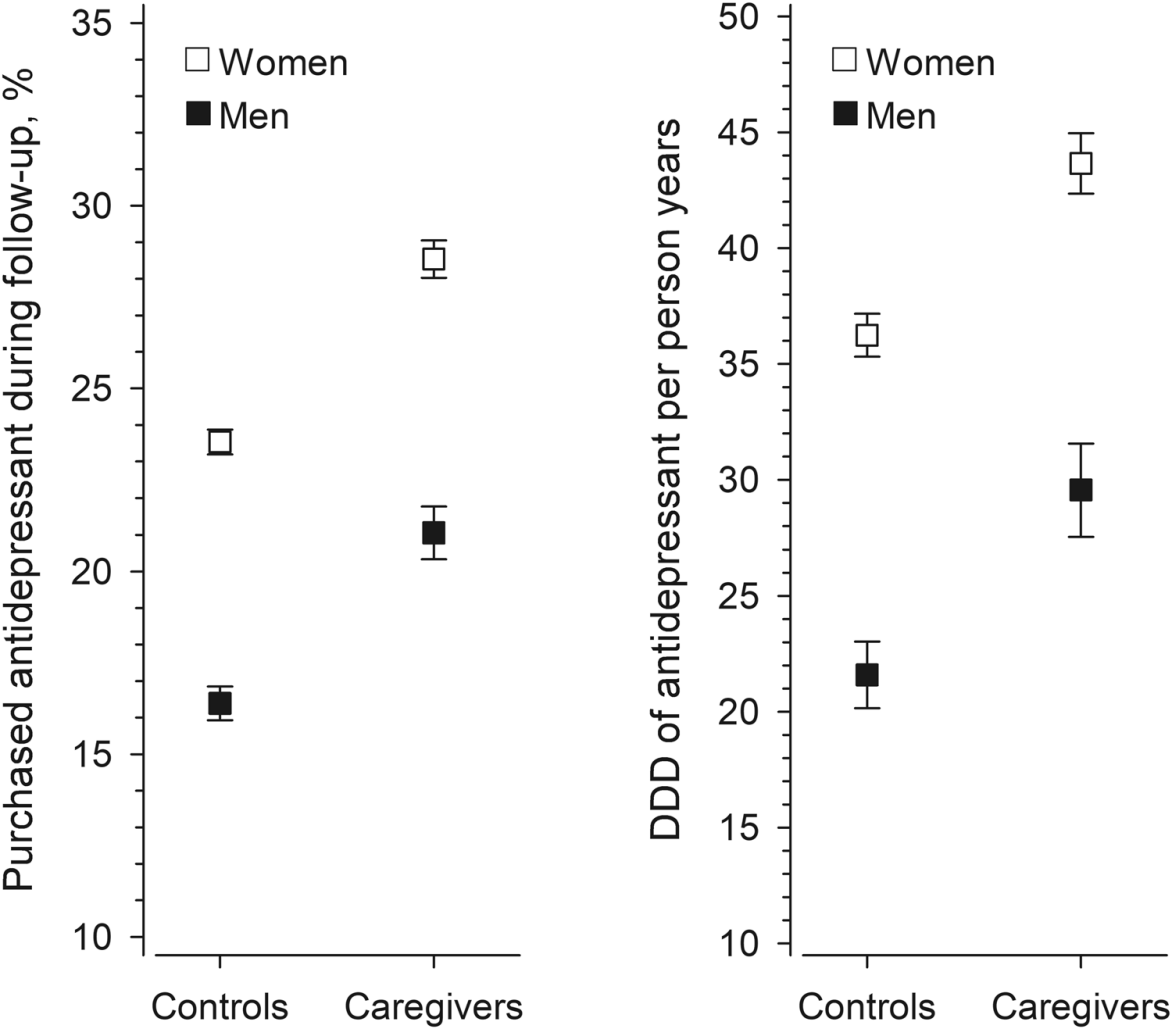
**a** Proportions of users of antidepressants and 95% confidence intervals adjusted for socioeconomic status during the 6-year follow-up. **b** Numbers of defined daily doses (DDD) of antidepressants used per person-year and 95% confidence intervals adjusted for socioeconomic status during the follow-up

Figure 1a presents the SES-adjusted proportions of those who used antidepressants during the 6-year follow-up. Both male and female caregivers were more likely to use antidepressants than their controls. Among women, 28.5% (95% confidence interval 28.0–29.1%) of caregivers and 23.5% (23.2–23.9%) of the controls used antidepressants during the follow-up. Among men, 21.1% (20.3–21.8%) of caregivers and 16.4% (15.9–16.9%) of the controls used antidepressants during the follow-up. The overall relative risk of having used antidepressants during the follow-up, adjusted for SES, was 1.21 (95% CI 1.18–1.24) for female caregivers as compared to female controls and 1.28 (95% CI 1.23–1.34) for male caregivers as compared to male controls. The numbers of daily doses used per person-year adjusted for SES were also higher in caregivers than in their controls (Fig. 1b). During the follow-up, female caregivers used 43.7 (42.4–45.0) and their controls used 36.2 (35.3–37.2) daily doses per person-year. Male caregivers used 29.6 (27.6–31.6) and their controls used 21.6 (20.2–23.0) daily doses per person-year.

**Fig. 2:**
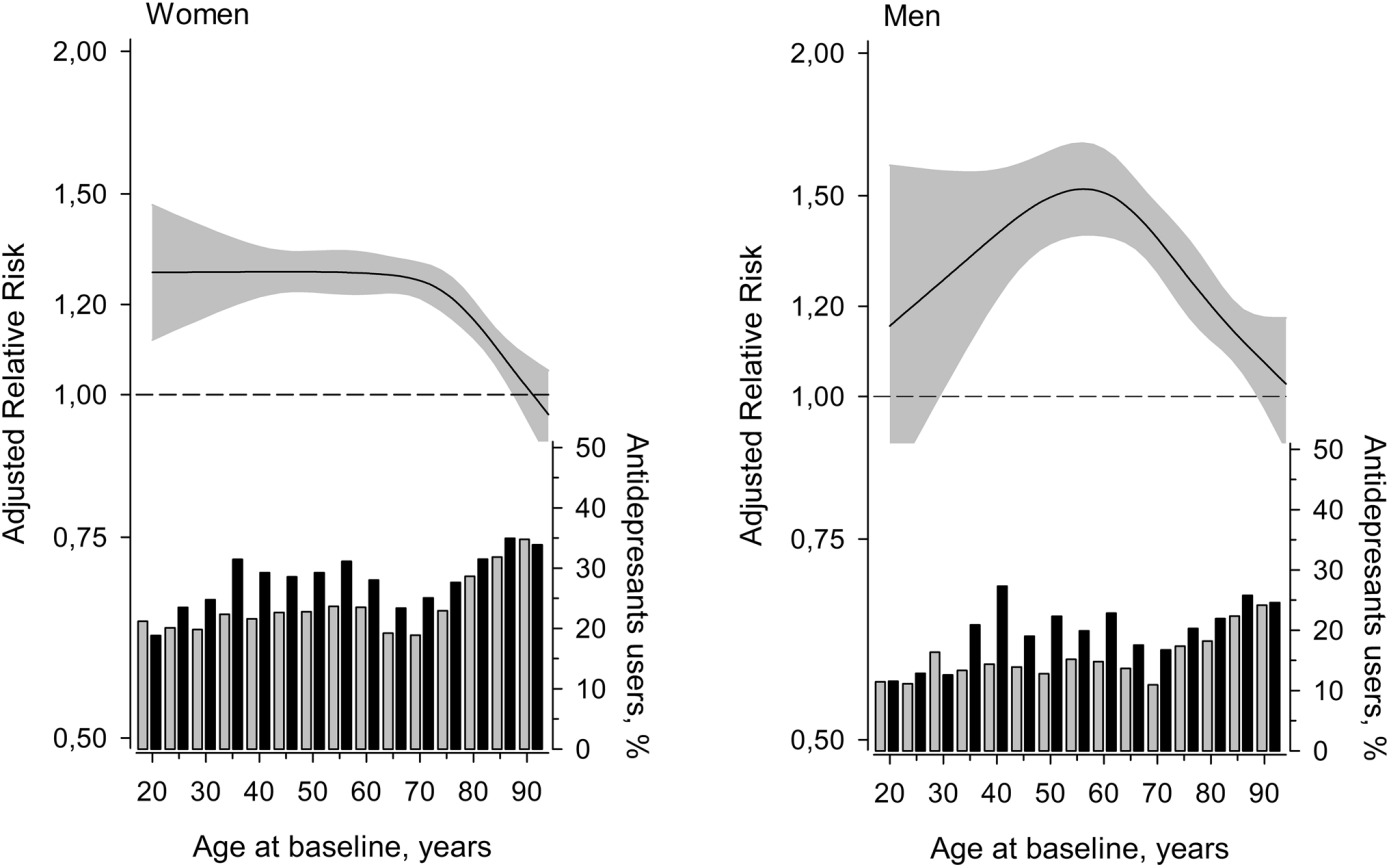
Line diagrams: relative risk of having used antidepressants for family caregivers compared to controls according to age at baseline (vertical axis on the left). The curves were derived from a 4-knot restricted cubic splines logistic regression models. The model was adjusted for socioeconomic status. The continuous lines show the relative risk estimate and the shaded area shows 95% confidence intervals. Bar diagrams: Proportions of antidepressant users for controls (grey bars) and caregivers (black bars) according to age in 5-year categories (vertical axis on the right)

Relative risk of having used antidepressant was also modelled as a function of age at baseline (Fig. 2). In both men and women, the risk of having purchased antidepressants adjusted for SES was higher in caregivers than in controls in all age groups. In female caregivers the relative risk was similar (about 1.3) from 20 to 70 years, after which the relative risk declined. In male caregivers, the relative risk was highest (about 1.4–1.5) between 45 and 65 years of age. The bar chart in Fig. 2 shows the probability of antidepressant use according to age category. Noteworthy is the observed increase in antidepressant use after the age of 70 years. This increase was steeper in the control group, narrowing down the differences between caregivers and controls in the oldest age groups.

## Discussion

We observed that family caregivers, who provided high-intensity care, were more likely to use antidepressants than their age-matched controls across most age groups. However, the risk of having used antidepressants among caregivers compared to controls varied as a function of age and this pattern was different in male and female caregivers.

Previous population-based surveys among caregivers have suggested that self-reported mental health of family caregivers is poorer than that in their non-caregiving peers [11, 15, 16]. However, some studies suggest that mental health is poorer only in caregivers who provide high-intensity care and better in those with lower-intensity care responsibilities [18, 19]. The results of our register-based study employing an objective outcome measure, antidepressant use, are in line with these previous findings. In the present study, officially recognized Finnish family caregivers, who can be considered as proving high-intensity care, were overall more likely to use antidepressants than the age-matched control population. Among both men and women, family caregivers also purchased a higher number of daily doses of antidepressants than the control population. Findings from a previous register-based study assessing the likelihood of being prescribed antidepressants among caregivers support our findings [19].

The analyses of the present study revealed variation in the relative risk of antidepressant use by age and sex. Among both men and women, caregivers had a higher likelihood for use of antidepressants than the controls up to age 85 years. However, in female caregivers, the likelihood of use of antidepressants was similar (relative risk about 1.3) from young adulthood up to 70 years of age and declined thereafter. In male caregivers, the likelihood of antidepressant use in relation to controls peaked (relative risk about 1.5) at middle age, between 45 and 65 years. A study based on Northern Ireland Census analysed the effect of age on antidepressant prescriptions in high-intensity caregivers (50+ h per week) compared to non-full-time caregivers, i.e., those providing less intensive care and non-caregivers combined [19]. The analysis suggested that relative to non-full-time caregivers, the youngest high-intensity caregivers (under 40 years) had the highest risk for being prescribed antidepressants, while the oldest high-intensity caregivers (age 70+ years) had the lowest risk. The analysis combined both sexes and merged all 70+ age groups, whereas our analysis showed marked variation in the probability of use of antidepressants in both caregivers and controls and in the relative risk of caregivers in age groups 70 years and above. The proportion of users of antidepressants increased markedly after age 70 years, and this increase was steeper in the controls. A plausible explanation for the increase with age in general and for the steeper increase in the older controls is proximity of death. Use of antidepressants increases with approaching death, possibly as a result of depressive symptoms related to multiple health problems at the end of life [26], and the older controls of this study were closer to death than the caregivers, as reported previously [27]. Hence, use of antidepressants among older caregivers relative to the controls is likely to be underestimated because of the difference in proximity of death between caregivers and controls.

Although the relative risk for use of antidepressants among male caregivers was higher compared to the controls in the present study, the overall level of use of antidepressants was higher among women than among men. This is in line with the generally higher prevalence of depression in women than in men although the ratio was smaller than the often reported 2:1 ratio [28, 29]. The amounts of antidepressants purchased by male caregivers and controls correspond to 4 and 3 week use per year, respectively, while the numbers among female caregivers and controls correspond to 6 and 5 week use per year, respectively. However, it should be noted that the majority of the subjects were older adults and they are often prescribed antidepressants with lower doses than the DDD [30]. Furthermore, for some indications, other than major depression, antidepressants can be prescribed with doses lower than the DDD.

The higher use of antidepressants in family caregivers may follow from stress originating in the high demands of caregiving. Long-term stress and stressful life events increase the risk of depression [31]. Furthermore, Pearlin’s model of caregiver stress suggests that apart from the demands of caregiving, stressors following from caregiving, such as economic problems and constriction of social life, may lead to depression and anxiety [3]. Caregivers may also struggle to manage between caregiving and other responsibilities and may be forced to give up other interests because of caregiving. The excess risk of antidepressant use associated with caregiving was particularly high among middle-aged men. It is possible that men find a caregiver identity very distant, because caregiving has traditionally been seen as a women’s task [32, 33] and because work may strongly be linked to their male identity [33]. Incongruence between an identity based on work and a caregiver identity may cause distress until the standards for these identities are adjusted [34]. It is also possible that male caregivers do not get social support as much as female caregivers. For example, peer support groups and other psychosocial support may be more suited for women’s than for men’s needs and preferences, because the majority of caregivers are women.

The strengths of this study include the large sample, including all officially recognized family caregivers in Finland and a matched control population. Completeness and accuracy of pharmacy records in the Nordic countries are considered to be high, higher than those based on use of medical records or surveys [35]. Since the vast majority of antidepressant purchases are covered by the reimbursement system the data can be considered to be comprehensive and representative at population level. The length of the follow-up, the large sample size, and the wide age range made it possible to analyse the effects of sex and age. A limitation in the study is that the reimbursement register does not contain information on the indications for which the antidepressants had been prescribed. Besides depression, some of the antidepressants have other indications, such as pain, anxiety, and sleep problems [36]. In older subjects, use of DDD as an estimate of the daily dose may underestimate the actual number of doses of antidepressants used. A lack of detailed information on the caregivers, care recipients and caregiving relationships, for example duration of caregiving or health condition of the care recipient, prevented further analyses on factors affecting antidepressant use in caregivers.

## Data Availability

The data that support the findings of this study are available from Finnish Tax Administration, and Findata but restrictions apply to the availability of these data, which were used under license for the current study, and so are not publicly available. Data are, however, available from the authors upon reasonable request and with permission of Finnish Tax Administration, and Findata.
